# The Effects of Puerarin on Rat Ventricular Myocytes and the Potential Mechanism

**DOI:** 10.1038/srep35475

**Published:** 2016-10-20

**Authors:** Hao Xu, Manxi Zhao, Shenghui Liang, Quanshu Huang, Yunchuan Xiao, Liang Ye, Qinyi Wang, Longmei He, Lanxiang Ma, Hua Zhang, Li Zhang, Hui Jiang, Xiao Ke, Yuchun Gu

**Affiliations:** 1 Laboratory of Molecular Pharmacology, IMM, Peking University, Beijing, 100871, China; 2 Laboratory of Innovative Drug Development and Translational Medicine Research of Traditional Chinese Medicine, Chengdu Kanghong Pharmaceutical Limited Company, Chengdu, 610036, China; 3 Department of Clinical Laboratory, Shaanxi Provincial Hospital of Traditional Chinese Medicine, Xi’an, 710003, China; 4 Department of Cardiology, Shaanxi Provincial Corps Hospital of Chinese People’s Armed Police Forces, Xi’an, 710054, China; 5 First Department of Surgery, Shaanxi Provincial Corps Hospital of Chinese People’s Armed Police Forces, Xi’an, 710054, China; 6 3rd affiliated hospital of Beijing University, Beijing, 100191, China

## Abstract

Puerarin, a known isoflavone, is commonly found as a Chinese herb medicine. It is widely used in China to treat cardiac diseases such as angina, cardiac infarction and arrhythmia. However, its cardioprotective mechanism remains unclear. In this study, puerarin significantly prolonged ventricular action potential duration (APD) with a dosage dependent manner in the micromolar range on isolated rat ventricular myocytes. However, submicromolar puerarin had no effect on resting membrane potential (RMP), action potential amplitude (APA) and maximal velocity of depolarization (Vmax) of action potential. Only above the concentration of 10 mM, puerarin exhibited more aggressive effect on action potential, and shifted RMP to the positive direction. Millimolar concentrations of puerarin significantly inhibited inward rectified K^+^ channels in a dosage dependent manner, and exhibited bigger effects upon Kir2.1 vs Kir2.3 in transfected HEK293 cells. As low as micromolar range concentrations of puerarin significantly inhibited Kv7.1 and IKs. These inhibitory effects may due to the direct inhibition of puerarin upon channels not via the PKA-dependent pathway. These results provided direct preclinical evidence that puerarin prolonged APD via its inhibitory effect upon Kv7.1 and IKs, contributing to a better understanding the mechanism of puerarin cardioprotection in the treatment of cardiovascular diseases.

The kudzu root (*Pueraria lobata*) have been used as a Chinese herbal medicines to treat cardiovascular diseases for thousand years. Puerarin, a known isoflavone, was commonly found as an important component in the kudzu root. It is the 8-c-glucoside of daidzein^1^ and has been widely prescribed in clinic in China to treat cardiac diseases such as heart failure, ischemia, angina pectoris^2^,^3^, cardiac infarction and arrhythmia^4^,^5^,^6^. Previously, patients, diagnosised with arrhythmia and manifested as premature ventricular/atrial contraction and ventricular tachycardia, were enrolled in a randomized, double-blind and placebo-controlled trials^7^. Puerarin group received intravenous injection on day 1 (at a dose of 200 mg puerarin, and 20 ml of 50% glucose solution over a period of 10 minutes), and on days 2 through 7 (10 mg per kilogram per day in 500 ml of 5% glucose solution). Both the attack frequency and the disappearance time of arrhythmia in puerarin group were significantly reduced compared with placebo group. Puerarin was also applied in clinic to treat arrhythmia with the advantage of this long-lasting effect^8^.

However, the mechanism of this anti-arrhythmia effect of puerarin remains unclear and less study is performed to illustrate the detailed mechanism of puerarin effect on cardiac electrical signal transmission. Puerarin was previously found to inhibit phosphatidylinositol 3-kinase (PI3 K)/protein kinase B (Akt) and c-jun-NH_2_-kinase (JNK) signaling pathways to retard the progress of cardiac dependent NADPH oxidase activation and mediated downstream redox-sensiutive-AP-1 signaling pathways to achieve its anti-hypertrophic effect^9^. Additionally, several studies revealed that puerarin antagonized ouabain-induced ventricular tachycardia in guinea pigs and chloroform/epinephrine-induced cardiac arrhythmias in rabbits^10^,^11^. Moreover, puerarin inhibited cardiac Na^+^ channel by shifting the steady-state inactivation curve without alternation in the shape of the I-V curve of INa^12^. Puerarin was also suggested to target upon P2X(3) to block the nociceptive of pain transmission which closely related with complications after myocardial ischemic injury and cardiac arrhythmia^13^.

Therefore, in this study, we concentrate upon the effect of puerarin on cardiac action potential and related ion channels in ventricles.

## Results

### Puerarin significantly prolonged APD of rat ventricular Nmyocytes

**Figure 1:**
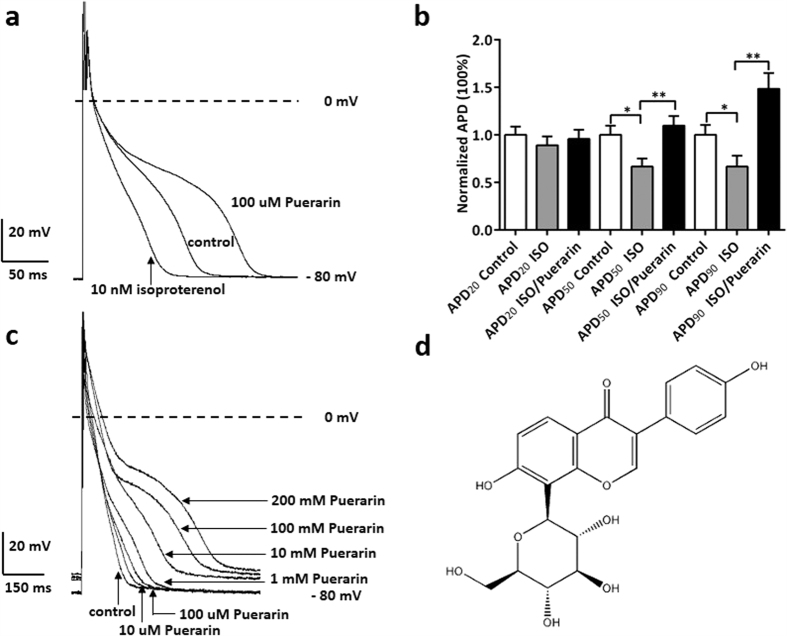
Puerarin prolonged APD of rat ventricular myocytes. (**a**) Puerarin at the concentrations of 100 μM significantly prolonged APD of rat ventricles; whereas isoproterenol at the concentrations of 10 nM significantly shortened APD of rat ventricles, in comparing with control. (**b**) Puerarin (100 μM) significantly offseted the reduced effect of isoproterenol (10 nM) and extended APD of rat ventricles at the 50% and 90% measurement points by respectively. *P < 0.01, **P < 0.001 versus control. n = 5 each point. (**c**) Puerarin at the concentrations of 10 μM, 100 μM, 1 mM, 10 mM, 100 mM and 200 mM extended APD of rat ventricles, in comparing with control. The statistical results were shown in table1. (**d**) The molecular structural formula of puerarin.

Puerarin is known as an isoflavone (Fig. 1d) that is found in a number of plants and herbs such as the root of pueraria^14^. Puerarin at the concentrations of 100 μM significantly prolonged APD of rat ventricular myocytes; whereas isoproterenol (10 nM) significantly shortened APD of rat ventricles, comparing with control (Fig. 1a,b). This prolonged APD was augmented when different concentrations of puerarin was applied, exhibiting a dosage dependent manner (Fig. 1c). Statistically, puerarin (100 μM) significantly prolonged APD_50_ from (71.8 ± 11.8) mS to (100.5 ± 14.1) mS and APD_90_ from (164.6 ± 21.4) mS to (221.6 ± 25.7) mS (Table 1); whereas RMP, APA and Vmax were not affected by application of puerarin. However, when 1 mM, 100 mM and 200 mM puerarin was applied, RMP was affected from −(80.1 ± 2.7) mV to −(75.3 ± 2.4) mV, −(74.7 ± 2.6) mV and −(73.5 ± 2.1) mV, respectively. The effect of the other dosages such as 10 μM and 1 mM of puerarin on ventricular action potential was also summarized in Table 1.

The results above indicated that puerarin apparently affected the repolarization phase of action potential, which are majorly contributed by IKs (slow delayed rectified, constituted by KvLQT1/MinK), IKr (rapid delay rectified, constituted by HERG)^15^ and IK1 (inward rectified, constituted by Kir2.x). To avoid the interference elicited by non-interested ion channels, suspected puerarin targeting ion channels such as inward rectified potassium channels, KvLQT1/Kv7.1 channels and IKs (KCNQ and KCNE) channels were therefore cloned and expressed in HEK cells^16^. The work below was performed in these expression systems.

### Puerarin at the concentration of milomolar range inhibited Kir 2.1 and Kir2.3 channels in a dosage dependent manner

**Figure 2:**
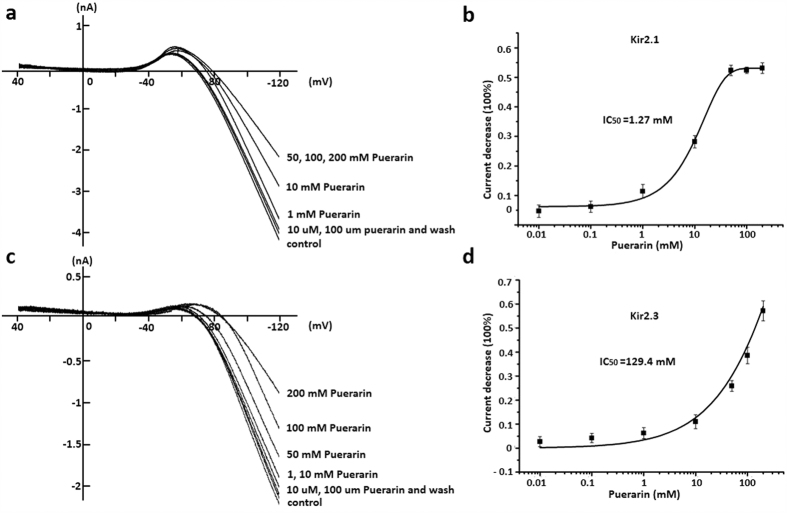
Puerarin inhibited Kir2.1 and Kir2.3 channels in a dosage dependent manner. (**a**) Puerarin at the concentrations of 10 μM, 100 μM, 10 mM, 50 mM, 100 mM, 200 mM significantly inhibited Kir2.1 currents illustrated by the ramp voltage protocol, respectively. This effect could be partly reversed by simple compound wash-off. (**b**) Concentration dependent inhibition by puerarin upon Kir2.1. The maximal inhibitory effect was (53.11 ± 1.84) % and saturation dosage was 50 mM. Dosage curve was fitted with non-linear Hill. IC_50_ = 1.27 mM, Hill coefficient is 0.990. n = 5 each point. (**c**) Puerarin at the concentrations of 10 μM, 100 μM, 1 mM, 10 mM, 50 mM, 100 mM, 200 mM significantly inhibited Kir2.3 currents illustrated by the ramp voltage protocol, respectively. This effect could be partly reversed by simple compound wash-off. (**d**) Concentration dependent inhibition by puerarin upon Kir2.3. Dosage curve was fitted with non-linear Hill. IC_50_ = 129.4 mM, Hill coefficient is 0.984. n = 5 each point.

The specificity of Kir2.1 was initially confirmed by its voltage-dependent Cs^+^ blockage^17^ and Ba^2+^ blockage (data not shown). The ramp voltage was a classic protocol to clearly observe the inward rectification of K^+^ current below its reverse potential^18^. Puerarin at the concentrations of 10 mM, 50 mM, 100 mM, 200 mM significantly inhibited Kir2.1 currents illustrated by the ramp voltage protocol, respectively. This effect could be partly reversed by washing-off compound (Fig. 2a). While puerarin at low concentrations of 10 μM, 10 μM, 1 mM had little effect on Kir2.1 currents. Additionally, there was a concentration dependent inhibition by puerarin upon Kir 2.1. The maximal inhibitory effect was (53.11 ± 1.84) % and saturation dosage was 50 mM. Dosage curve was fitted with non-linear Hill. IC_50_ = 1.27 mM, Hill coefficient is 0.990 (n = 5 each point) (Fig. 2b).

Human Kir2.2 Kir2.3 and Kir2.1 (KCNJ12, KCNJ4 and KCNJ2^19^,^20^,^21^, constitute human myocardial I_Kir_. Kir2.3 channels were expressed in HEK-293 cells to further clarify the effect of puerarin on Kir2. Puerarin at the concentrations of 10 mM, 50 mM, 100 mM, 200 mM significantly inhibited Kir2.3 currents illustrated by the ramp voltage protocol, respectively. This effect could also be reversed by washing-off compound (Fig. 2c). However, puerarin at low concentrations of 10 μM, 100 μM, 1 mM also had less effect on Kir2.3 currents. Additionally, a concentration dependent inhibition by puerarin upon Kir 2.3 was also shown in Fig. 2c. Dosage curve was fitted with non-linear Hill. IC_50_ = 129.4 mM, Hill coefficient was 0.984. n = 5 each point (Fig. 2d). However, it is noticed that the effective inhibitory dosage of puerarin is extremely high and it was rarely to use in clinic^22^.

### Puerarin at the concentration of micromolar range inhibited Kv7.1 channels in a dosage dependent manner

Kv7.1 was assembled by KCNQ1, which is the α-subunit of a voltage-dependent potassium channel. Co-assembly KCNQ1 with an auxiliary β subunit KCNE1 form IKs^23^. The biophysical properties of KCNQ1 was notably modified by KCNE1, including slowing activation and deactivation kinetics, increasing unitary channel conductance, shifting the voltage dependence of activation to more negative potentials^24^. Abundant intracellular molecules and pathways, including cAMP, ATP, tyrosine kinases, protein kinase A (PKA) are reported to regulate Kv7.1/IKs^25^.

**Figure 3:**
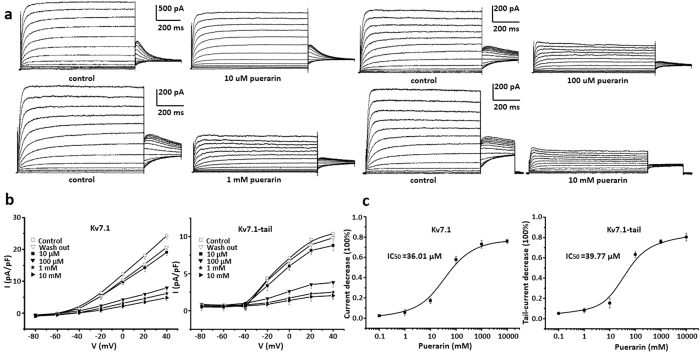
Puerarin inhibited Kv7.1 channels in a dosage dependent manner. (**a**) Puerarin at the concentrations of 10 μM, 100 μM, 1 mM and10 mM significantly inhibited Kv7.1 currents illustrated by the protocol of voltage steps, respectively. (**b**) I-V curves summarized inhibitory effects of puerarin at concentrations of 10 μM, 100 μM, 1 mM, 10 mM and recovery after compound wash off. Different concentrations of puerarin decreased the Kv7.1 from (24.3 ± 1.1) pA/pF to (19.1 ± 1.0) pA/pF, (7.9 ± 0.8) pA/pF, (6.1 ± 0.4) pA/pF, (4.8 ± 0.8) pA/pF, and decreased the Kv7.1-tail current from (10.4 ± 0.6) pA/pF to (8.8 ± 0.7) pA/pF, (3.8 ± 0.6) pA/pF, (2.5 ± 0.9) pA/pF, (2.0 ± 0.5) pA/pF at 40 mV, respectively. (**c**) Concentration dependent inhibition by puerarin upon Kv7.1 at the peak currents and tail currents. Dosage curve was fitted with non-linear Hill. IC_50_ = 36.01 μM at the peak currents and IC_50_ = 39.77 μM at the tail currents, Hill coefficient is 0.997. n = 6 each point.

Because the tail current was one of the perspectives of Kv7.1^26^, the specific protocol based on the voltage steps was majorly used in this study. Puerarin at the concentrations of 10 μM, 100 μM, 1 mM and 10 mM significantly inhibited Kv7.1 currents, respectively (Fig. 3a). I-V curves summarized inhibitory effects of 10 μM, 100 μM, 1 mM, 10 mM puerarin upon Kv7.1. This inhibitory effect could be reversed by washing-off compound (Fig. 3b). There was a concentration dependent inhibition by puerarin upon Kv7.1 at the both peak currents and tail currents (Fig. 3c). Dosage curve was fitted with non-linear Hill. IC_50_ = 36.01 μM at the peak currents and IC_50_ = 39.77 μM at the tail currents, Hill coefficient was 0.997. n = 6 each point.

### Puerarin inhibited IKs channels in a dosage dependent manner

**Figure 4:**
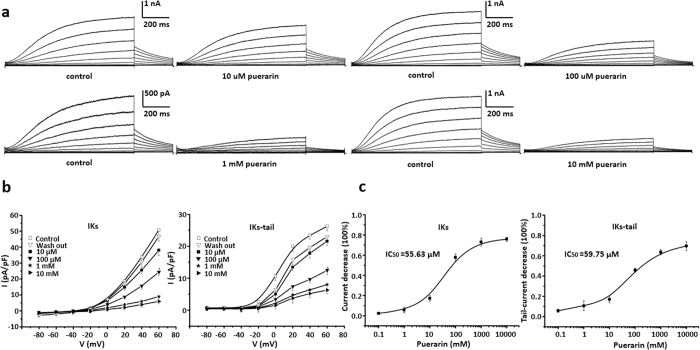
Puerarin inhibited IKs channels in a dosage dependent manner. (**a**) Puerarin at the concentrations of 10 μM, 100 μM, 1 mM and10 mM significantly inhibited IKs currents illustrated by the protocol of voltage steps, respectively. (**b**) I-V curves summarized inhibitory effects of puerarin at concentrations of 10 μM, 100 μM, 1 mM, 10 mM and recovery after compound wash off. Different concentrations of puerarin decreased the IKs from (33.8 ± 3.2) pA/pF to (25.6 ± 2.4) pA/pF, (15.7 ± 1.9) pA/pF, (6.0 ± 1.3) pA/pF, (5.4 ± 1.7) pA/pF, and decreased the IKs-tail current from (23.5 ± 1.2) pA/pF to (17.8 ± 1.5) pA/pF, (9.7 ± 1.1) pA/pF, (6.4 ± 1.2) pA/pF, (5.2 ± 1.3) pA/pF at 40 mV, respectively. (**c**) Concentration dependent inhibition by puerarin upon IKs at the peak currents and tail currents. Dosage curve was fitted with non-linear Hill. IC_50_ = 55.63 μM at the peak currents and IC_50_ = 59.75 μM at the tail currents, Hill coefficient is 0.989. n = 6 each point.

IKs currents were majorly mediated by channels composed of KCNQ and KCNE^27^. Puerarin at the concentrations of 10 μM, 100 μM, 1 mM and 10 mM significantly inhibited IKs currents, respectively (Fig. 4a). I-V curves summarized inhibitory effects of 10 μM, 100 μM, 1 mM, 10 mM puerarin upon IKs. This inhibitory effect could be reversed by washing-off compound (Fig. 4b). A concentration dependent inhibition by puerarin upon IKs at the peak currents and tail currents were show in Fig. 4c. Dosage curve was fitted with non-linear Hill. IC_50_ = 55.63 μM at the peak currents and IC_50_ = 59.75 μM at the tail currents, Hill coefficient was 0.989. n = 6 each point.

The results above clearly suggested that puerarin inhibitory effect upon Kv7.1 and IKs was in the range of micromolar concentration, which was achievable in clinic application. Inhibitory effects of puerarin upon Kir2.1 and Kir 2.3 required a concentration in hundreds millimolar and it was rare in clinic application. The following mechanism study was then focused on the Kv7.1 and IKs.

### Puerarin might inhibit the current of Kv7.1/IKs in a PKA-independent pathway

**Figure 5:**
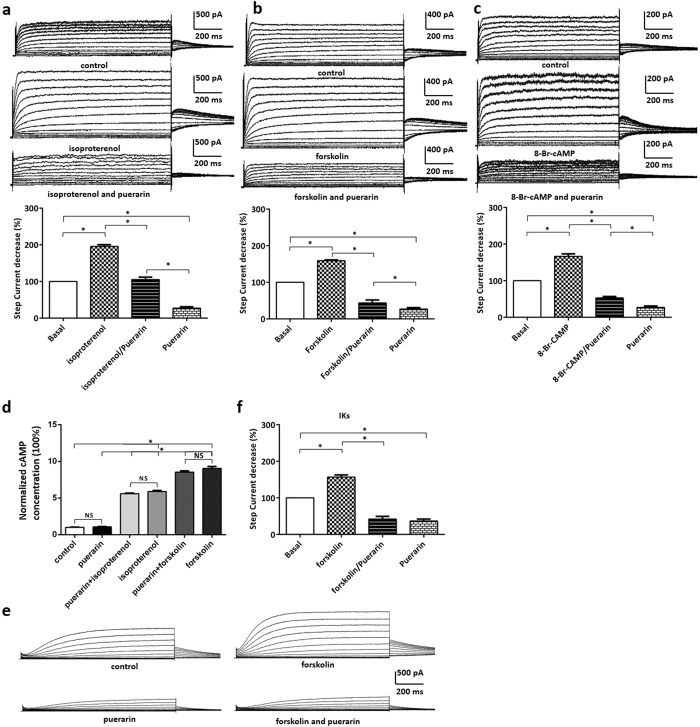
Puerarin might inhibit the current of Kv7.1/IKs in a PKA-independent pathway. (**a**) Isoproterenol significantly enhanced Kv7.1 current whereas puerarin (100 μM) antagonized such activation effect. (**b**) Forskolin significantly enhanced Kv7.1 current whereas puerarin (100 μM) antagonized such activation effect. (**c**) 8-Br-cAMP significantly enhanced Kv7.1 current whereas puerarin (100 μM) antagonized such activation effect. (**d**) 1 mM puerarin had no effect on intracellular cAMP concentration and no effect upon the increase of cAMP induced by isoproterenol, forskolin in rat cardiac myocytes. (**e**) forskolin significantly enhanced IKs current whereas puerarin (100 μM) antagonized such activation effect in rat cardiac myocytes. (**f** ) Statistical analysis of Fig. 5e.

It is well known the activation of G protein-coupled receptors (GPCR) could augment Kv7.1 and IKs via a PKA pathway^28^. Isoproterenol was an agonist of β1 receptor which couples with GPCR^29^. Application of isoproterenol activated adenylyl cyclase which producted cAMP. Alternatively, forskolin activated adenylyl cyclase directly to generate cAMP^30^. Application of isoproterenol, forskolin and 8-Br-cAMP consistently augmented Kv7.1 and IKs current in HEK293 cells (Fig. 5a–c). Puerarin reversed these augment effects and furtherly inhibited Kv7.1 currents (Fig. 5a–c). The additional results revealled that puerarin at the concentration of 1 mM had no effect on intracellular cAMP concentration both in HEK293 transfected Kv7.1/IKs channel and in rat cardiac myocytes (Fig. 5d). Suggesting that puerarin had no effect upon the increase of cAMP induced by isoproterenol and forskolin. In addition, we confirmed the results in the rat ventricular myocytes that puerarin significantly inhibited IKs despite pharmacological activation of PKA with forskolin (Fig. 5e,f). Overall, these results partly illustrated that puerarin might inhibit the current of Kv7.1/IKs in a PKA-independent manner and suggested puerarin might principally and directly inhibit channels.

### Puerarin might directly inhibit Kv7.1 and IKs channels

**Figure 6:**
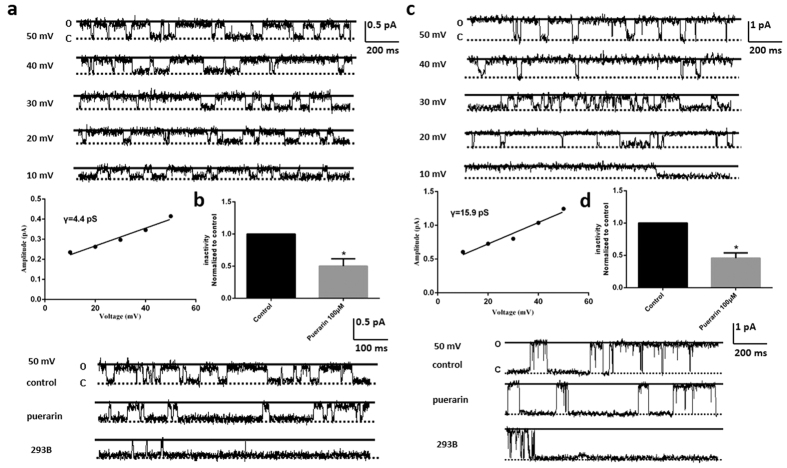
Puerarin might directly inhibited Kv7.1 and IKs channels. (**a**) Single channel currents with the conductance of 4.4 pS elicited at the different voltages. (**b**) The open probability of Kv7.1 channel was reduced by puerarin at the concentration of 100 μM significantly, which was further abolished by the specific antagonist of Kv7.1, 293B. (**c**) Single channel currents with the conductance of 15.9 pS elicited at the different voltages. (**d**) The open probability of IKs channel was reduced by puerarin at the concentration of 100 μM significantly, which was further abolished by the specific antagonist of IKs, 293B.

In the inside-out recording model, puerarin was applied directly to the intracellular side of ion channels and dissociated of puerarin in activities due to cellular barrier limitations. Single channel currents of Kv7.1 possessed the conductance of 4.4 pS elicited at the different voltages (Fig. 6a). The open probability of Kv7.1 channel was significantly reduced by puerarin at the concentration of 100 μM, which was further abolished by a specific antagonist of Kv7.1, 293B (Fig. 6b).

Single channel currents of IKs possessed the conductance of 15.9 pS elicited at the different voltages (Fig. 6c). The open probability of IKs channel was also reduced by puerarin at the concentration of 100 μM significantly, which was also further abolished by a specific antagonist of IKs, 293B (Fig. 6d).

### Baicalin inhibited Kir 2.1 channels, whereas enhanced IKs channels in a dosage dependent manner

**Figure 7:**
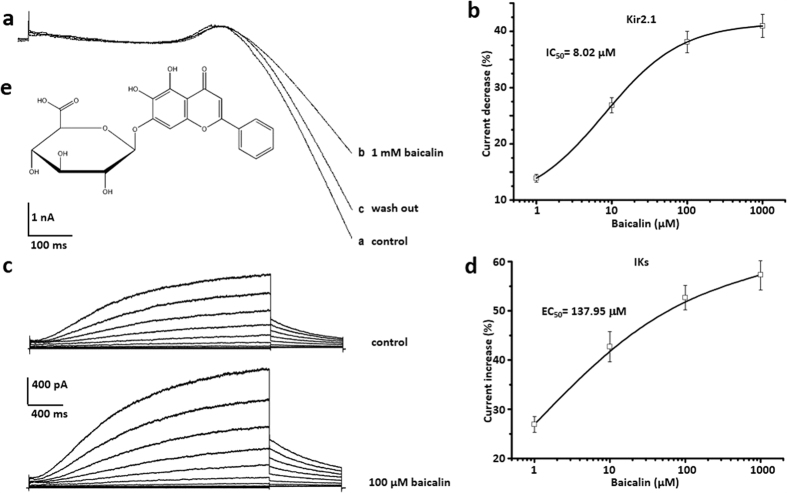
Baicalin inhibited Kir 2.1 channels and enhanced IKs channels in a dosage dependent manner. (**a**) Baicalin at the concentrations of 1 mM significantly inhibited Kir2.1 currents illustrated by the ramp voltage protocol. This effect could be partly reversed by simple compound wash-off. (**b**) Concentration dependent inhibition by baicalin upon Kir 2.1. The maximal inhibitory effect was (40.95 ± 2.05) % and saturation dosage was 1 mM. Dosage curve was fitted with non-linear Hill. IC_50_ = 8.02 μM, Hill coefficient is 0.983. n = 5 each point. (**c**) Baicalin at the concentrations of 100 μM significantly enhanced IKs currents illustrated by the protocol of voltage steps, respectively. (**d**) Concentration dependent enhancement by baicalin upon IKs currents. The maximal increased effect was (57.32 ± 3.07) % and saturation dosage was 1 mM. Dosage curve was fitted with non-linear Hill. EC_50_ = 137.95 μM at the peak currents, Hill coefficient is 0.993. n = 6 each point. (**e**) The molecular structural formula of baicalin.

Baicalin was known as a flavone (Fig. 7e) and was found in plants as scutellaria baicalensis^31^ and herbs as HUANGQIN^32^. In contrast to puerarin, Baicalin at the concentrations of 1 mM significantly inhibited Kir2.1 currents illustrated by the ramp voltage protocol, and this effect could be reversed by washing-off compound (Fig. 7a). There is a concentration dependent inhibition by baicalin upon Kir 2.1. The maximal inhibitory effect was (40.95 ± 2.05) % and saturation dosage was 50 mM. Dosage curve was fitted with non-linear Hill. IC_50_ = 8.02 μM, Hill coefficient is 0.983 (n = 5 each point) (Fig. 7b). In addition, baicalin at the concentrations of 100 μM significantly increased IKs illustrated by the voltage steps protocol (Fig. 7c), and this effect could be reversed by washing-off compound (data not shown). There was a concentration dependent enhancement by baicalin upon IKs currents. The maximal increased effect was (57.32 ± 3.07) % and saturation dosage was 1 mM. Dosage curve was fitted with non-linear Hill. EC_50_ = 137.95 μM, Hill coefficient was 0.993 (n = 5 each point) (Fig. 7d).

## Discussion

In this study, puerarin significantly prolonged ventricular APD when the concentration as low as micromolar range and exhibited a dosage dependent inhibitory manner. However, submicromolar puerarin had no effect on resting RMP, APA and Vmax of action potential. Only above the concentration of 10 mM, Puerarin exhibited more aggressive effect on action potential, and shifted RMP to the positive direction. In addition, as low as micromolar range of concentrations, puerarin significantly inhibited Kv7.1 and IKs. These inhibitory effects were due to the directly inhibition of puerarin upon channels rather than the PKA pathway. In the millimolar range of concentrations, puerarin significantly inhibited inward rectified K^+^ channels, and exhibited bigger effect upon Kir2.1 vs Kir 2.3. These results provided direct evidence and mechanism that puerarin prolonged APD majorly via its inhibitory effect upon Kv7.1 and IKs, supporting the widely clinical application of puerarin in treatment of cardiovascular diseases.

We also demonstrated that puerarin could reverse 10 nM ISO-mediated AP shortening on rat ventricular myocytes. Besides this cellular model of arrhythmia, several studies had announced puerarin could accelerate cardiac angiogenesis and improve cardiac function of myocardial infarction rats by upregulating VEGFA, Ang-1 and Ang-2^33^. Another study demonstrated that puerarin improved cardiac function after MI in diabetic mice through increasing the expression and translocation of GLU4 while attenuating the expression and translocation of CD36^34^.

In addition to previous studies, which suggested that puerarin affected calcium-activated potassium channels^35^ and fast Na^+^ channels^12^, our results revealed that puerarin significantly influences Kv7.1, IKs and IK1. According to the change of action potential by puerarin, the extension of APD majorly contributed to its pharmacology perspectives. Although puerarin exerted inhibitory effects upon Kir2.1, Kir2.3, Kv7.1 and IKs, it exhibited distinct effective dosage, IC_50_ and saturation. At a micromolar concentration, puerarin inhibited KvLQT1 and IKs and when application of milimolar puerarin, it affected Kir2.1, Kir2.3, KvLQT1 and IKs. Prolonged APD played a key role in slowing down heart rate and lengthening the refractory period of myocytes, these results might explain possible mechanism of action in the treatment of arrhythmia. Very recent comprehensive analysis of regional ion channel expression in normal and myopathic hearts documented that the ratio among Kir2.1, Kir2.2 and Kir2.3 was roughly around 13:7:8 in normal left ventricles and around 16:5:13 in myopathic left ventricles^36^. Such similar alternations in expression of K_V_7.1/IKs in the procession of myopathic path might also occur, illustrating distinct pharmacology effects of puerarin in different ion channel expression profiles in this physiol-pathological process. In different patients, puerarin might exhibit different treatment efficiency due to the cardiac profiles with different ion channel composition.

In the heart, IKs had an important role in cardiac action potential repolarization^37^. IKs contributed little to the ventricular action potential repolarization under normal circumstances^38^. However, IKs was markedly improved when ventricular repolarization faced challenging conditions, such as increased sympathetic tone. The slow activation and deactivation kinetics led to accumulation of IKs, maintaining channel open state. Thus, under challenging situations, IKs prevented excessive action potential prolongation and development of arrhythmogenic early afterdepolarization by providing a repolarization reserve^39^,^40^.

To date, nearly three hundred mutations in KCNQ1 and at least 20 mutations in KCNE1 have been identified, casing long QT syndrome (LQT), short QT syndrome (SQT), sinus bradycardia and atrial fibrillation (AF)^41^. Most of the mutations caused a loss of function of KCNQ1 and KCNE1, resulting in LQTS1 and LQT5 syndrome, an inherited disorder characterized by a delayed ventricular repolarization, syncope and sudden death^42^. However, a few gain-of-function mutations identified in KCNQ1 and KCNE1 were associated with atrial fibrillation and/or the SQT syndrome^43^. Although it still remains unclear puerarin affects the conclusive domain or binding site of the channels (such as basolateral targeting signal, PKA phosphorylation site and A-kinase anchoring protein binding), we are aware that further site-mutation studies of vital domain or binding site are needed to reveal the underlining mechanism. In this study, our results partly revealed that puerarin probably inhibit KCNQ1 and IKs via direct inhibition, as puerarin was applied directly to the intracellular side and reduced the open probability of the channels under the inside-out recording model. Modulation of KCNQ1 and KCNE1 might be the mechanism of puerarin in the clinic treatment of arrhythmia.

Our results were consistent to a previous study in the conclusion that puerarin could inhibit inward rectified potassium channels^44^, but inconsistent with a different effective dosage range. This discrepancy was mainly attributed to the different components and expressions of ionic currents in interspecies in ventricular myocytes. Besides, rat ventricular action potentials were considerably shorter in duration than that of guinea pig, which had a well-defined plateau phase. The rat ventricular myocytes exhibited a large initial phase of repolarization which was rapid at depolarized potentials, and was slower at negative potentials^45^. In our system, expression systems were employed to simplify the condition and avoid the interference. Our results suggested that puerarin could affect inward potassium channels but in an extremely high dosage. Such dosage was rarely used in clinic. Kir2.3 exhibited more sensitive to puerarin in contrast to Kir2.1, suggesting puerarin might compete with PIP_2_ to bind with Kir.

Interestingly, baicalin is a flavone, an analog of puerarin but extracted from another Chinese medicinal herb named *Scutellaria baicalensis*^32^. In a similar concentration range, Baicalin was found to inhibit Kir2.1 but augment IKs (Fig. 7). Such distinct effects reflected their different clinic results and vivided pharmacological effects from herbs. A recent study^46^ revealed the effect of baicalin on producing mesenteric arteries relaxation by activating large-conductance Ca^2+^ -activated K^+^ channel (BK(Ca)) and inhibiting voltage-dependent Ca^2+^ channel (VDCC) in a concentration-dependent manner. The cGMP/PKG and cAMP/PKA pathways had been known in the regulation of these two channels. Therefore, further study was necessary to confirm the additional and disparate effect between puerarin and baicalin on cardiac arrhythmia.

In summary, the present study provides the novel inhibitory effect of puerarin on Kv7.1/IKs underlining the mechanism of extending APD and anti-arrhythmic activity. Puerarin may directly inhibit Kv7.1/IKs channel independent of PKA activation, and it certainly inhibits Kir2.1 and Kir2.3 channels in some extent, suggesting the rational and discreet application of puerarin in clinic treatment of cardiovascular diseases.

## Methods

### Chemicals

Puerarin was obtained from Beijing Saisheng Pharmaceutial Company (98.4% purity), taurine, K_2_-ATP, HEPES, EGTA, baicalin, isoproterenol, forskolin, the cAMP analog 8-Bromoadenosine-3′, 5′-cyclic monophosphorothioate were purchased from Sigma-Aldrich (St. Louis., MO, USA). Collagenase (type II) and bovine serum albumin (BSA) were purchased from Invitrogen (Carlsbad, CA, USA). All other reagents were of analytical grade. All drugs and reagents were dissolved in distilled water unless otherwise noted. Isoproterenol (1 mM) was dissolved in 0.1% ascorbic acid in distilled water. Baicalin (10 mM), forskolin (100 mM) was dissolved in DMSO. Final DMSO concentrations did not exceed 0.1%.

### Animal experiments

Fifty male Sprague-Dawley rats (10–12 weeks) were used for this experiment. Myocytes were prepared from ventricles by standard collagenase dissociation technique. All animals received humane care in compliance Guide for the Institutional Animal Care and Use Committee (IACUC) and was approved by the Ethics Review Board for Animal Studies of Institute of Molecular Medicine (IMM), Peking University). All methods were carried out in accordance with the approved guidelines.

### Solutions

The Tyrode’s solution contained (mM): 126 NaCl, 5.4 KCl, 1.8 CaCl_2_, 1 MgCl_2_, 0.33 NaH_2_PO_4_, 10 HEPES and 10 glucose, pH 7.4 (adjusted with NaOH). The Ca^2+^-free Tyrode’s solution was prepared by removing CaCl_2_ from the Tyrode’s solution. The Kreb’s solution used for cell storage contained (mM): 70 glutamic acid, 30 KCl, 15 taurine, 0.5 MgCl_2_, 10 KH_2_PO4, 0.5 EGTA, 10 HEPES, 10 glucose, and 1% albumin, pH 7.4 (adjusted with KOH). The enzyme solution used for the rat cardiomyocyte isolation contained 0.08 g/L collagenase (type II) and 1.0 g/L BSA in Kreb’s solution. For action potential recording on cardiomyocyte, the pipette solution contained (mM): 100 potassium aspartate, 30 KCl, 1 MgCl_2_, 5 Mg-ATP, 5 EGTA, 5 HEPES, pH 7.3 (adjusted with KOH); the Tyrode’s solution was used as the standard bath solution. For whole-cell recording of IKs on myocytes, the pipette solution contained (mM): 125 potassium aspartate, 20 KCl, 1 MgCl_2_, 5 Mg-ATP, 10 EGTA, 5 HEPES, pH 7.3 (adjusted with KOH); the bath solution contained (mM): 132 NaCl, 4 KCl, 1.8 CaCl_2_, 1.2 MgCl_2_, 10 HEPES, 5 glucose, pH 7.4 (adjusted with NaOH). 3 μM nifedipine and 5 μM E-4031 were used to block the calcium current and IKr, respectively. For whole-cell recording on HEK-293 cells, the pipette solution contained (mM): 140 KCl, 2 MgCl_2_, 10 HEPES, 0.1 EGTA, 4 K_2_-ATP, pH 7.3 (adjusted with KOH); the bath solution contained (mM): 120 NaCl, 5 KCl, 1.5 CaCl_2_, 1 MgCl_2_, 10 HEPES, 10 glucose, pH 7.4 (adjusted with NaOH). For single-channel recording, the pipette solution (extracellular) contained (mM): 145 KCl, 10 HEPES, 10 glucose, pH 7.4 (adjusted with KOH); the bath solution (intracellular) contained (mM): 145 KCl, 1.2 MgCl_2_, 10 HEPES, 0.1 EGTA, pH 7.38 (adjusted with KOH).

### Cell preparations

Single ventricular myocytes were isolated from adult rat hearts via enzymatic dissociation as previously described^47^. Briefly, the rats were anesthetised with 25% chloral hydrate (0.1 g/100 g). Their hearts were excised rapidly and retrogradely perfused on a Langendorff apparatus with a Ca^2+^-free Tyrode’s solution for 5 min before the perfusate was switched to an enzymatic solution for 10–20 min. The perfusate was finally changed to Kreb’s solution for 5 min. The perfusates were bubbled with 95% O2 + 5% CO2 and maintained at 37 °C. The ventricles w ere cut into small chunks and gently agitated in Kreb’s solution. The cells were filtered through nylon mesh (pore size 200 μm) and stored in Kreb’s solution at 4 °C.

### Recording of action potentials

Action potentials were elicited at 1 Hz by 2 ms current pulses applied using the patch pipette. The APD at 50% and 90% repolarization (APD_50_ and APD_90_), RMP, APA and Vmax were measured.

### Whole cell patch clamp recording

Cell suspensions were transferred into a chamber on the stage of a Nikon Eclipse Ti inverted microscope (Tokyo, Japan). The whole-cell recordings were performed using isolated single ventricular myocytes. Patch electrodes were pulled from a horizontal microelectrode puller (P-1000, Sutter Instrument Co, USA) and fire polished to final tip resistance of 4.0–8.0 MΩ when filled with internal solutions. Signals were amplified with an Axopatch 200B amplifier (Axon Instruments, USA) and filtered at 1 kHz. The pCLAMP 9.0 software was used to generate current and voltage clamp protocols, acquire data, and analyse the current traces. The whole-cell series resistance was compensated to more than 80%. All of the experiments were performed at room temperature (22 ± 1 °C). The whole-cell IK1 was elicited by a ramp voltage protocol from −140 to + 40 mV in 1 s, or at a 0 mV holding potential to test potentials from −140 to + 40 mV in 10 mV increments. The IK1 was calculated as the difference between the peak inward current and the holding current level. The whole-cell Kv7.1/IKs were elicited from a −30 mV holding potential to test potentials from −80 to + 40 mV in 10 mV increments.

### Single-channel inside-out patch clamp recording

Single-channel activity was recorded at a series of holding levels ranging from 10 mV of 50 mV. The data were acquired at 20 KHz and lowpass filtered at 5 kHz. During post-analysis, data were further filtered at 300 Hz. Single-channel events were listed and analysed by pclampfit 9.0 (single-channel search-in-analyse function). NPo, the product of the number of channels and the open probability, was used to measure the channel activity within a patch. In total, 50% threshold cross-method was used to determine valid channel openings. Initial (1–2 min) single-channel records were normally used as the control. The activity of Kv7.1/IKs during application of chemicals was normalized to activity during the control period to assess the effects of chemicals on Kv7.1/IKs activity.

### cDNA constructs

Human Kir2.1 cDNA, Kir2.3 cDNA, Kv7.1 cDNA and KCNE1 cDNA, was cloned in Human Embryonic Kidney 293 cells, respectively. In some cases, Kv7.1 cDNA and KCNE1 cDNA were simultaneously cloned in Human Embryonic Kidney 293 cells.

All of the cDNAs above were confirmed with DNA sequencing (Majorbio, Shanghai, China).

### Mouse/Rat cAMP assay

cAMP levels were measured in rat cardiomyocytes using a Mouse/Rat cAMP kit (cAMP parameter assay kit, R&D Systems) according to the manufacturer’s instructions. The assay is based on the competitive binding technique. A streptavidin-coated plate is incubated with a biotinylated monoclonal antibody specific for cAMP. Following a wash to remove excess monoclonal antibody, cAMP present in a sample competes with a fixed amount of horseradish peroxidase (HRP)-labeled cAMP for sites on the monoclonal antibody. This is followed by another wash to remove excess conjugate and unbound sample. A substrate solution is added to the wells to determine the bound enzyme activity. The color development is stopped and the absorbance is read at 450 nm. The intensity of the color is inversely proportional to the concentration of cAMP in the sample. The level of cAMP in the sample was determined based on a standard curve and expressed as pmol/mg per each sample. Normalized cAMP was calculated by compared with the control group. In our study, we investigate the effect of puerarin on intracellular cAMP concentration and its effect upon the increase of cAMP induced by isoproterenol, forskolin and 8-Br-cAMP.

### Statistics

The data were presented as the Mean ± SD. Dosage curves were fitted with pCLAMP 9.0 (Axon Instruments) and software Origin 7.0 (OriginLab, USA). The statistical significance was determined using a t-test when comparing two groups and ANOVA when comparing multiple groups. A value of P < 0.05 was considered statistically significant.

## Additional Information

**How to cite this article**: Xu, H. *et al*. The Effects of Puerarin on Rat Ventricular Myocytes and the Potential Mechanism. *Sci. Rep.* **6**, 35475; doi: 10.1038/srep35475 (2016).

## Figures and Tables

**Table 1:** Effects of different concentrations of puerarin on action potential properties of rat ventricular myocytes.

Group	RMP/mV	APA/mV	V_max_ V/s	APD_50_/ms	APD_90_/ms
Control	−80.1 ± 2.7	112.5 ± 6.3	226.9 ± 12.4	71.8 ± 11.8	164.6 ± 21.4
Puerarin (mM)					
0.01	−80.5 ± 2.2	109.8 ± 8.5	217.5 ± 10.7	86.9 ± 10.7^*^	188.3 ± 11.5^*^
0.1	−80.3 ± 1.6	114.2 ± 4.9	242.1 ± 13.8	100.5 ± 14.1^#^	221.6 ± 25.7^#^
1	−79.7 ± 1.5	115.9 ± 6.2	236.1 ± 15.9	123.6 ± 25.4^#^	278.7 ± 28.2^#^
10	−75.3 ± 2.4^#^	112.6 ± 4.7	238.4 ± 11.5	131.4 ± 19.6^*^	330.1 ± 22.3^*^
100	−74.7 ± 2.6^#^	113.8 ± 5.1	240.6 ± 12.1	146.9 ± 21.8^#^	415.6 ± 28.4^#^
200	−73.5 ± 2.1^#^	111.7 ± 5.6	237.8 ± 10.4	162.3 ± 23.5^#^	439.5 ± 29.6^#^

Note: RMP, resting membrane potential; APA, action potential amplitude; Vmax, maximal velocity of depolarization; APD_50_ and APD_90_, the APD at 50% and 90% repolarization. *P < 0.05, ^#^P < 0.01 *vs* the control group, respectively.

## References

[b1] Overstreet D. H. . NPI-031 G (puerarin) reduces anxiogenic effects of alcohol withdrawal or benzodiazepine inverse or 5-HT2 C agonists. Pharmacol Biochem Behav 75, 619–625 (2003). 12895679 10.1016/s0091-3057(03)00114-x

[b2] Gao Z., Wei B. & Qian C. Puerarin injection for treatment of unstable angina pectoris: a meta-analysis and systematic review. Int J Clin Exp Med 8, 14577–14594 (2015). 26628941 PMC4658830

[b3] Wang Q. . Puerarin injection for unstable angina pectoris. Cochrane Database Syst Rev CD004196 (2006). 16856037 10.1002/14651858.CD004196.pub2

[b4] Gao Q. . Atractyloside and 5-hydroxydecanoate block the protective effect of puerarin in isolated rat heart. Life Sci 79, 217–224 (2006). 16458326 10.1016/j.lfs.2005.12.040

[b5] Yuan Y. . Puerarin attenuates pressure overload-induced cardiac hypertrophy. J Cardiol 63, 73–81 (2014). 23906530 10.1016/j.jjcc.2013.06.008

[b6] Takaishi K. & Watanabe Y. [Studies on the decoction of Chinese medicines. II. The extraction of some drugs by starch aqueous solution and the property of Gegen Tang]. Yakugaku Zasshi 91, 1092–1097 (1971). 5170012 10.1248/yakushi1947.91.10_1092

[b7] Luo Z. K., Liu Y. & Li H. M. [A clinical efficacy and safety study on coronary heart disease and angina treatment with Puerarin Injection]. Zhonghua Liu Xing Bing Xue Za Zhi 33, 614–616 (2012). 22883272

[b8] Fan L. L., Zhao D. H., Zhao M. Q. & Zeng G. Y. [The antidysrhythmic effect of puerariae isoflavones]. Yao Xue Xue Bao 20, 647–651 (1985). 3834744

[b9] Gang C. . Puerarin Suppresses Angiotensin II-Induced Cardiac Hypertrophy by Inhibiting NADPH Oxidase Activation and Oxidative Stress-Triggered AP-1 Signaling Pathways. J Pharm Pharm Sci 18, 235–248 (2015). 26158288 10.18433/j3n318

[b10] Chai X. S. . [Anti-arrhythmic action of puerarin]. Zhongguo Yao Li Xue Bao 6, 166–168 (1985). 2874687

[b11] Qian Y. . Blocking effect of puerarin on calcium channel in isolated guinea pig ventricular myocytes. Chin Med J (Engl) 112, 787–789 (1999). 11717945

[b12] Zhang G. Q. . Puerarin blocks Na+ current in rat ventricular myocytes. Acta Pharmacol Sin 24, 1212–1216 (2003). 14653946

[b13] Liang S., Xu C., Li G. & Gao Y. P2X receptors and modulation of pain transmission: focus on effects of drugs and compounds used in traditional Chinese medicine. Neurochem Int 57, 705–712 (2010). 20863868 10.1016/j.neuint.2010.09.004

[b14] Hayakawa J., Noda N., Yamada S. & Uno K. [Studies on physical and chemical quality evaluation of crude drug preparations. I. Analysis of Pueraria Radix and species Puerariae]. Yakugaku Zasshi 104, 50–56 (1984). 6737221 10.1248/yakushi1947.104.1_50

[b15] Bryant S. M., Wan X., Shipsey S. J. & Hart G. Regional differences in the delayed rectifier current (IKr and IKs) contribute to the differences in action potential duration in basal left ventricular myocytes in guinea-pig. Cardiovasc Res 40, 322–331 (1998). 9893726 10.1016/s0008-6363(98)00133-3

[b16] Ukomadu C., Zhou J., Sigworth F. J. & Agnew W. S. muI Na+ channels expressed transiently in human embryonic kidney cells: biochemical and biophysical properties. Neuron 8, 663–676 (1992). 1314619 10.1016/0896-6273(92)90088-u

[b17] Thuringer D., Lauribe P. & Escande D. A hyperpolarization-activated inward current in human myocardial cells. J Mol Cell Cardiol 24, 451–455 (1992). 1378902 10.1016/0022-2828(92)91833-q

[b18] Delmar M., Ibarra J., Davidenko J., Lorente P. & Jalife J. Dynamics of the background outward current of single guinea pig ventricular myocytes. Ionic mechanisms of hysteresis in cardiac cells. Circ Res 69, 1316–1326 (1991). 1934360 10.1161/01.res.69.5.1316

[b19] Kaibara M. . Identification of human Kir2.2 (KCNJ12) gene encoding functional inward rectifier potassium channel in both mammalian cells and Xenopus oocytes. FEBS Lett 531, 250–254 (2002). 12417321 10.1016/s0014-5793(02)03512-3

[b20] Hassinen M., Paajanen V., Haverinen J., Eronen H. & Vornanen M. Cloning and expression of cardiac Kir2.1 and Kir2.2 channels in thermally acclimated rainbow trout. Am J Physiol Regul Integr Comp Physiol 292, R2328–R2339 (2007). 17289820 10.1152/ajpregu.00354.2006

[b21] Zobel C. . Molecular dissection of the inward rectifier potassium current (IK1) in rabbit cardiomyocytes: evidence for heteromeric co-assembly of Kir2.1 and Kir2.2. J Physiol 550, 365–372 (2003). 12794173 10.1113/jphysiol.2002.036400 PMC2343053

[b22] Jiang R. W. . A comparative study on aqueous root extracts of Pueraria thomsonii and Pueraria lobata by antioxidant assay and HPLC fingerprint analysis. J Ethnopharmacol 96, 133–138 (2005). 15588661 10.1016/j.jep.2004.08.029

[b23] Abbott G. W. KCNE1 and KCNE3: The yin and yang of voltage-gated K channel regulation. Gene (2015). 10.1016/j.gene.2015.09.059 PMC4917010 26410412

[b24] Hu Y. . Engineering a peptide inhibitor towards the KCNQ1/KCNE1 potassium channel (IKs). Peptides 71, 77–83 (2015). 26188173 10.1016/j.peptides.2015.07.002

[b25] Matavel A. & Lopes C. M. PKC activation and PIP(2) depletion underlie biphasic regulation of IKs by Gq-coupled receptors. J Mol Cell Cardiol 46, 704–712 (2009). 19233191 10.1016/j.yjmcc.2009.02.006 PMC2668609

[b26] Howard R. J., Clark K. A., Holton J. M. & Minor D. L. Jr. Structural insight into KCNQ (Kv7) channel assembly and channelopathy. Neuron 53, 663–675 (2007). 17329207 10.1016/j.neuron.2007.02.010 PMC3011230

[b27] Melman Y. F., Krummerman A. & McDonald T. V. KCNE regulation of KvLQT1 channels: structure-function correlates. Trends Cardiovasc Med 12, 182–187 (2002). 12069759 10.1016/s1050-1738(02)00158-5

[b28] Xie Y., Grandi E., Puglisi J. L., Sato D. & Bers D. M. beta-adrenergic stimulation activates early afterdepolarizations transiently via kinetic mismatch of PKA targets. J Mol Cell Cardiol 58, 153–161 (2013). 23481579 10.1016/j.yjmcc.2013.02.009 PMC3628092

[b29] Wang S. . Adrenergic regulation of the rapid component of delayed rectifier K+ currents in guinea pig cardiomyocytes. J Thorac Dis 6, 1778–1784 (2014). 25589973 10.3978/j.issn.2072-1439.2014.12.27 PMC4283310

[b30] Gilissen J. . Forskolin-free cAMP assay for G-coupled receptors. Biochem Pharmacol (2015). 10.1016/j.bcp.2015.09.010 26386312

[b31] Zhou Y. P. & Zhang J. Q. Oral baicalin and liquid extract of licorice reduce sorbitol levels in red blood cell of diabetic rats. Chin Med J (Engl) 102, 203–206 (1989). 2527143

[b32] Huang Y. . Baicalin-induced vascular response in rat mesenteric artery: role of endothelial nitric oxide. Clin Exp Pharmacol Physiol 29, 721–724 (2002). 12100008 10.1046/j.1440-1681.2002.03706.x

[b33] Ai F. . Puerarin accelerate scardiac angiogenesis and improves cardiac function of myocardial infarction by upregulating VEGFA, Ang-1 and Ang-2 in rats. Int J Clin Exp Med 8, 20821–20828 (2015). 26885006 PMC4723851

[b34] Cheng W. . Puerarin improves cardiac function through regulation of energy metabolism in Streptozotocin-Nicotinamide induced diabetic mice after myocardial infarction. Biochem Biophys Res Commun 463, 1108–1114 (2015). 26079885 10.1016/j.bbrc.2015.06.067

[b35] Guo X. G., Chen J. Z., Zhang X. & Xia Q. [Effect of puerarin on L-type calcium channel in isolated rat ventricular myocytes]. Zhongguo Zhong Yao Za Zhi 29, 248–251 (2004). 15706853

[b36] Sivagangabalan G. . Regional ion channel gene expression heterogeneity and ventricular fibrillation dynamics in human hearts. PLoS One 9, e82179 (2014). 24427266 10.1371/journal.pone.0082179 PMC3888386

[b37] Agsten M. . BACE1 modulates gating of KCNQ1 (Kv7.1) and cardiac delayed rectifier KCNQ1/KCNE1 (I). J Mol Cell Cardiol (2015). 10.1016/j.yjmcc.2015.10.006 26454161

[b38] Dvir M., Peretz A., Haitin Y. & Attali B. Recent molecular insights from mutated IKS channels in cardiac arrhythmia. Curr Opin Pharmacol 15, 74–82 (2014). 24721657 10.1016/j.coph.2013.12.004

[b39] Lee H. C. . Modulation of KCNQ1 alternative splicing regulates cardiac IKs and action potential repolarization. Heart Rhythm 10, 1220–1228 (2013). 23608591 10.1016/j.hrthm.2013.04.014 PMC3771516

[b40] Jost N., Papp J. G. & Varro A. Slow delayed rectifier potassium current (IKs) and the repolarization reserve. Ann Noninvasive Electrocardiol 12, 64–78 (2007). 17286653 10.1111/j.1542-474X.2007.00140.x PMC6931982

[b41] Qureshi S. F. . Novel mutations of KCNQ1 in Long QT syndrome. Indian Heart J 65, 552–560 (2013). 24206879 10.1016/j.ihj.2013.08.025 PMC3861163

[b42] Bartos D. C. . A KCNQ1 mutation contributes to the concealed type 1 long QT phenotype by limiting the Kv7.1 channel conformational changes associated with protein kinase A phosphorylation. Heart Rhythm 11, 459–468 (2014). 24269949 10.1016/j.hrthm.2013.11.021 PMC4333640

[b43] Moreno C. . A new KCNQ1 mutation at the S5 segment that impairs its association with KCNE1 is responsible for short QT syndrome. Cardiovasc Res 107, 613–623 (2015). 26168993 10.1093/cvr/cvv196

[b44] Zhang H. . Puerarin: a novel antagonist to inward rectifier potassium channel (IK1). Mol Cell Biochem 352, 117–123 (2011). 21327545 10.1007/s11010-011-0746-0

[b45] Varro A., Lathrop D. A., Hester S. B., Nanasi P. P. & Papp J. G. Ionic currents and action potentials in rabbit, rat, and guinea pig ventricular myocytes. Basic Res Cardiol 88, 93–102 (1993). 8389123 10.1007/BF00798257

[b46] Lin Y. L. . Baicalin, a flavonoid from Scutellaria baicalensis Georgi, activates large-conductance Ca2+ -activated K+ channels via cyclic nucleotide-dependent protein kinases in mesenteric artery. Phytomedicine 17, 760–770 (2010). 20171070 10.1016/j.phymed.2010.01.003

[b47] Grosso D. S., Frangakis C. J., Carlson E. C. & Bressler R. Isolation and characterization of myocytes from the adult rat heart. Prep Biochem 7, 383–401 (1977). 918020 10.1080/00327487708061656

